# Paternal and Maternal Variables Related to Depression in Childhood

**DOI:** 10.3390/ijerph17010275

**Published:** 2019-12-31

**Authors:** Antonio Raya Trenas, Beatriz Aguilar Yamuza, Javier Herruzo Cabrera, María J. Pino Osuna

**Affiliations:** Department of Psychology. University of Córdoba, 14005 Córdoba, Spain; beatris.ay@hotmail.com (B.A.Y.); ed1hecaf@uco.es (J.H.C.); ed1piosm@uco.es (M.J.P.O.)

**Keywords:** depression, family relations, child rearing, parenting, logistic models

## Abstract

Several studies have highlighted the relationship between parenting styles and depression in children. The aim of this study is to ascertain whether there are differences in the parenting practices received by two groups of children who obtain low-risk and high-risk scores respectively in relation to depression and determine which parenting variables are linked with the presence or absence of this kind of internalizing problem. From a sample of 550 subjects, obtained by probabilistic cluster sampling, we selected 140 children between 3 and 12 years of age who met a set of specific criteria as having high risk scores (70 children) for depression according to the Behavior Assessment System for Children (BASC) or having low scores in this variable (70 children). Then, the Parent–Child Relationship Inventory (PCRI) was applied to both parents. We carried out a binomial logistic regression analysis which resulted in a prediction model for 89.3% of the sample, based on the following parenting variables: limit setting and involvement from the mothers, and parental support, autonomy, satisfaction with parenting, and communication from the fathers. Finally, the usefulness of our results to plan intervention strategies within families of children with depression is discussed.

## 1. Introduction

The Diagnostic and Statistical Manual of Mental Disorders, Fifth Edition (DSM-5) [1] defines feelings of sadness and hopelessness, crying, low self-esteem, irritable moods, difficulties in social relationships, loss of interest in daily activities, fatigue, trouble falling asleep, and increased or decreased appetite and weight, among other things, as characteristic symptoms of depression in children and adolescents. Of these different childhood disorders, it is the internalizing disorders which are more difficult to perceive and detect, since they are not visible in children’s observable behavior [2,3]. Furthermore, difficulties in identifying this type of disorder in children may partly be due to the scarcity of suitable instruments of evaluation. One of the few standardized instruments that are available is the Behavior Assessment System for Children (BASC) [3], an instrument that has been adapted for several countries, including Spain, and which can be used with young people between the ages of 3 and 18. Researchers into evaluation processes at these ages argue that information must be obtained in the environment in which the problem occurs, and this instrument allows data to be submitted by parents and tutors, who it considers the principal source of information in the early years of a child’s life [3,4]. According to Achenbach and Edelbrock [5], these internalizing disorders can be defined as a combination of environmental problems that the child exhibits through behaviors of anxiety, timidity, avoidance, nervousness, fears, sadness, preoccupation, among other symptoms.

The fact that childhood depression has acquired a relevant value in recent years as one of the main internalizing disorders is not coincidental. According to the World Health Organization (WHO) [6], depression is considered one of the most frequent mental health problems affecting work capacity and productivity. It is one of the principal causes of disability, with more than 300 million estimated sufferers throughout the world. The WHO [6] predicts that in 2020 depression will be the second cause of disability in the world. The results of a worldwide study have shown that the prevalence of depression in childhood is 2.6% [7]. In Spain, prevalence varies depending on the studies consulted, with rates ranging from 5% to 13% and rising with age [8]. Several studies coincide in pointing out that depression increases with age, showing higher prevalence in adolescence and adulthood [8,9]. Prevalence is higher in girls from the age of 12. Earlier, girls do not show significant differences with boys [10,11].

Several studies have highlighted the relationship between the influence of parenting styles and depression in children [12,13]. Parenting style is defined as the complex of behaviors and attitudes developed by parents towards their children in their daily interactions [14]. More specifically, certain types of parental behavior, such as neglect, rejection, criticism, excessive control, and overprotection, have been found to be more associated with depression in children [15,16,17]. Maternal behavior shows a direct relationship with the development of emotions in children [18]. In this regard, childhood depression can be determined by a lack of discipline, by excessively rigid discipline, by rejection, and by lack of love [10]. Zubizarreta, Calvete, and Hankin [19] point out that punishments received by children from their parents could increase their internalizing problems and, in particular, exacerbate their psychological symptoms. In contrast, communication between parents and children is associated with a lower risk of depression in the latter, since it strengthens relationships between the two by establishing bonds of union, respect for opinions and signs of mutual affection, and cultivating [20] parental warmth [19].

Other factors that indirectly affect depression in children are family conflicts [8,21] and, more specifically, higher levels of dysphoria (i.e., depressive moods or sadness) [22]. Lawrence et al. [23] point out that greater family disorganization leads to parenting practices based on negative discipline, contrary to what happens with parents who perceive satisfactory family interaction. In addition to disorganized family environments, inappropriate parenting styles and pessimistic styles are also associated with depression in children [17]. Richard de Minzi [24] highlights the difference between families with a democratic parenting style, who favor adaptive confrontations and have a protective effect vis-à-vis depression, and authoritarian families who do not. Another variable which may influence the development of depression in children is the mental and emotional state of the parents [25]. Wilkinson, Trzaskowski, Haworth, and Eley [26] point out that depressed parents are less likely to provide emotional support and affection for their children and more likely to instill negative or erroneous ideas in them. Del Barrio [8] concludes that 60% of depressed children had a mother with depressive symptoms. Paternal depression also influences children, affecting learning patterns, interactive stimulation, and genetic influence [27]. One study shows that the children of parents who suffered from a depressive disorder (in their childhood or youth) are three times more likely to initiate depression in childhood and seven times more likely to initiate depression in adulthood [28].

Most parents with children of this age do not employ a consistent parenting style, acting in some situations in a democratic way, in other situations in an authoritarian manner [29]. This instability is particularly characteristic of the caregiving style of some parents, whose discontinuous behavior can negatively impact the children’s tendency to depression, due to their inability to comprehend their parents’ behavior [30]. Several studies suggest that mothers are more supportive and protective of children, while fathers are more likely to encourage children’s participation with others [31,32]. The behavior of the mother and father in parenting affects children differently. The control a father can exert, for example, is more accepted by children, whereas conflict can emerge with the mother when children disagree with her maternal behavior.

Although the number of studies focusing on this problem is increasing in children, few studies consider parenting style relevant for predicting depression. It would be interesting to create predicting models for this disorder based on some characteristics of the parenting style of fathers and mothers. Such models would shine some light on possible patterns that could be established in order to improve the relations between parents and children and consequently, help preventing this type of disorder.

Ultimately, this study aims to ascertain whether there are differences in the parenting style received by two groups of children who score high and low risk respectively on the instrument used to measure depression and determine which parenting variables are linked with the presence or absence of this kind of internalizing problem. In other words, we expect to develop a predictive model for belonging to a group with high scores or to another group with low scores on depression, based on some parenting variables.

## 2. Materials and Methods 

### 2.1. Participants

An initial sample of 550 subjects was obtained by probabilistic cluster sampling. These children belonged to Nursery and Primary Schools of Andalusia (Spain). In this sample, the subjects were extracted from six schools: two schools were in the capital, two schools belonged to populations with more than 20,000 habitants, and two schools belonged to populations with less than 20,000 habitants. We selected a broad sample of participants from an average socioeconomic, they did not present any specific risk characteristics. After a preliminary assessment of the 550 subjects, 140 subjects were selected and divided into two groups of 70: the first group encompassed all participants situated in the high-risk group in terms of the variable depression as reported by their parents (risk group), having obtained a T score over 60, as discussed in greater detail in the description of the instrument below. The second group comprised all participants with a T score under 40 for this variable (low depression group), and for this selection process they were paired with those from the risk group in terms of gender and school level.

Both groups consisted of 39 boys and 31 girls aged from 3 to 12, with an average age of 7.19 (Standard Deviation (SD) = 2.911) in the high-risk group and 6.97 (SD = 2.988) in the low-scoring group for depression, with no significant differences between the two groups in relation to this variable, since t = −0.430 (*p* = 0.698). Each group was distributed as follows in terms of school level: 32 (46%) from Nursery Education, 7 (10%) from the first and second level of Primary Education, 12 (17%) from the third and fourth level of Primary Education, and 19 (27%) from the fifth and sixth level of Primary Education.

All subjects were treated according to the ethical rules of the American Psychological Association and gave their informed consent. The research ethics committee at Universidad de Cordoba approved the research procedure and certified that the project respected the main principles established by the Helsinki Declaration.

### 2.2. Instruments

In order to compile information, the following instruments were used:

An adaptation into Spanish of the Behavior Assessment System for Children (BASC) [3]. The purpose of this system is to evaluate a wide range of pathological and adaptive dimensions using different sources of information (parents, teachers, and children) and different methods (questionnaires, developmental history, and observation). In this case, the questionnaires for parents were used. These questionnaires, which are divided into three levels according to age (3–6, 6–12, 12–18), present an internal consistency index of 0.70. Test–retest correlation (three months interval) was 0.85, 0.88, and 0.70 for the three levels of questionnaire completed by parents. For this sample, the internal consistency index was 0.74.

Of all the different scales included in this instrument, the depression scale was used for this study, defined by the instrument as “feelings of unhappiness, sadness and stress that can result in an inability to carry out daily activities or can lead to thoughts of suicide”. This scale presents internal consistency indexes between 0.70 and 0.77, depending on the age of the subjects.

The scores obtained for any of the scales were transformed into T scores, which indicate the distance of a particular score in relation to the control group mean, thereby enabling comparisons to be made between subjects of different ages. These T scores can vary between 0 and 100 and present a mean of 50 and SD of 10. On the basis of these T scores, different levels are established: scores below 30 are considered very low, under 40 low, between 40 and 60 intermediate, over 60 at risk, and over 70 clinically significant.

The other instrument utilized was the Parenting Questionnaire (PCRI-M) by Roa and Del Barrio [33], adapted from the Parent–Child Relationship Inventory [34], which measures paternal and maternal practices and attitudes towards parenting using a direct score. It comprises 78 items with four response options (totally disagree, disagree, agree, and strongly agree), which are grouped into seven scales. High scores on the different scales indicate greater agreement with the situation defined in each scale. The seven scales are as follows:

Support, social and emotional, received by a mother or a father.
Satisfaction with parenting: satisfaction obtained by a parent through parenthood.
Involvement: level of interaction and parental knowledge about their child.
Communication: perception regarding the effectiveness of communication with their child.
Limit setting: level of exigency in obedience of rules.
Autonomy: ability to give the child independence.
Role orientation: attitudes about the role played by each gender in parenting.

A small social desirability scale is also included.

The internal consistency of the instrument for this sample, obtained using Cronbach’s alpha coefficient, was 0.87. For each scale, this coefficient ranged from 0.68 for the Support scale to 0.78 for the Satisfaction scale. Furthermore, in the case of this sample, the questionnaire presented good construct validity, given the correlations between the different scales on the questionnaire, especially in the most important parenting scales such as Involvement–Satisfaction with parenting (0.51), Involvement–Communication (0.64), Limit setting–Support (0.42), Limit setting–Autonomy (0.44), and Satisfaction with parenting–Limit setting (0.37).

### 2.3. Procedure

First of all, we contacted six Nursery and Primary Schools of Andalusia. Once the schools were selected as indicated in the Participants section, and their school board had consented to participate in the study, contact was made with the head teachers of the schools. After consent of head teachers of the schools, the questionnaire was passed to all families in the school. We got a response rate of 86%. To inform the families in writing of this study and provide them with the instrument, teachers collaborated in the distribution and collection of questionnaires. Families that agreed to collaborate voluntarily completed the P form (parents) of the BASC in its different versions depending on the age of their children, and the PCRI-M, completed by both father and mother, considering the lack of information from both parents an exclusion criteria.

### 2.4. Data Analysis

To evaluate the possible effect of parenting variables on depression, an ex post facto design was applied with a quasi-control group. Hence, a dichotomic variable was used as the dependent variable, derived from the T score obtained in depression. The two options for this variable were 0 for subjects with a low depression score and 1 for subjects in the risk area. For this purpose, subjects were selected if their T score in depression, as reported by their parents, situated them above the level of risk, and another group was chosen with low depression scores, equivalent to the first group in terms of gender and school level. 

Subsequently, a binary logistic regression analysis was performed [35] Based on the coefficients estimated by logistic regression for each of the variables, in accordance with its probability of belonging to either level of the dependent variable, this process classified each subject into one of the two categories proposed.

Logistic regression enabled various models to be established, the most efficient one being the model that predicts the highest percentage of correctly classified subjects with the lowest number of possible variables, since the main purpose of this analysis is to establish a model that predicts the dependent variable using the independent variables. The model comprises an equation made up of estimated coefficients and the scores of the different variables, giving a resulting score between 0 and 1, with a cut-off point of 0.5: scores between 0.5 and 1 indicate the probability of obtaining a high score in depression; scores between 0 and 0.5 indicate the contrary.

To perform these analyses, the following predictive variables were taken into account, from the perspective of both the father and the mother: Support, Satisfaction with parenting, Involvement, Communication, Limit setting, Autonomy, and Role Orientation.

## 3. Results

On the depression scale, for possible T scores between 0 and 100, the risk group obtained a mean T score of 69.10 (SD = 7.269), ranging from 60 to 86, whereas the low-depression group obtained a mean score of 36.69 (SD = 2.462), with a minimum of 30 and a maximum of 40.

With respect to the parenting style factors of mothers and fathers, mean scores obtained by risk group and the low-score group were compared through analysis of variance. As we can see in Table 1, the low-score group presents significantly higher mean scores on all factors.

By applying binary logistic regression analysis to these two groups, between the different possible models, a prediction model was established comprising the six factors described in Table 2. This six-factor model was selected since it predicts whether a subject will belong to one or the other group for a large percentage of the sample with a fairly small number of variables. All factors act positively, that is, increased support, satisfaction, communication, and autonomy of father decreases depression in children. Furthermore, this problem decreases when involvement and limit setting of mother are greater. The goodness-of-fit for the model was good, with a Chi-Square of 123.470 and six degrees of freedom, statistically different from zero. Furthermore, the Cox and Snell R-square and the Nagelkerke R-square presented good values: 0.586 and 0.781, respectively. The Hosmer–Lemeshow test to evaluate correspondence between real and predicted values of the dependent variable did not provide significant results, since *X*^2^ = 6.863 (*p* = 0.551).

As for the classification of subjects, a mean percentage of 89.3% was obtained for correctly classified subjects, obtaining small differences between the two groups: 88.6% for the risk group and 90% for the low-score group.

One of the main applications of logistic regression analysis is the possibility of creating an equation that can be used to classify a subject in one of the conditions of the dependent variable and knowing the probability of manifesting a high level of depression depending on the score obtained for one or more of the independent variables.

The equation is

b_1_ = 1/1 + e^−z^

where

Z = B_0_ + B_1_(X_1_) + B_2_(X_2_) + B_3_(X_3_) + B_n_(X_n_)

and

Z = linear combination of variables.
B_0_ = estimated coefficient of the constant regression.
B_1_ = estimated coefficient for variable 1.
X_1_ = subject’s score for variable 1.
b_1_ = probability of belonging to the risk group.
e = base of natural logarithms (2.718).

By transferring the data from the study to the equation described above, we get

Z = 45.780 + (−0.286)(X_1_) + (−0.171)(X_2_) + (−0.182)(X_3_) + (−0.223)(X_4_) + (−0.278)(X_5_) + (−0.251)(X_6_)

where

X_1_ = father’s score for support.
X_2_ = father’s score for satisfaction with parenting.
X_3_ = father’s score for communication.
X_4_ = father’s score for autonomy.
X_5_ = mother’s score for involvement.
X_6_ = mother’s score for limit setting.

Finally, the result obtained for Z is transferred to the first equation to obtain the probability of b_1_ or probability of obtaining a risk score in conduct problems.

**Figure 1:**
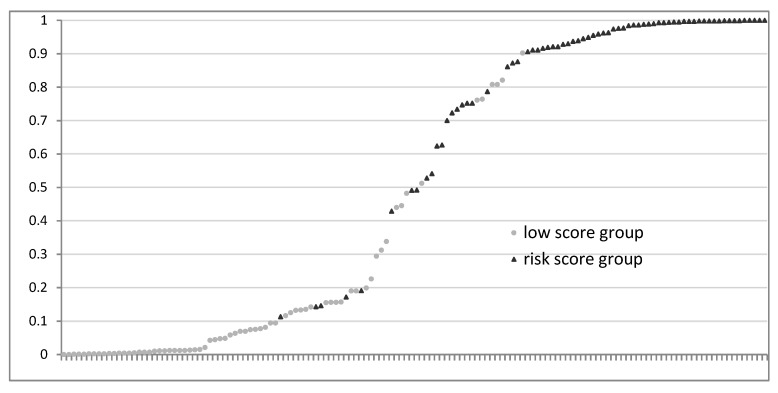
Observed groups and probability prediction.

When two subjects were chosen at random, one from the low-score group (subject number 25) and another from the risk group (subject number 97), the probability of b_1_ obtained for the first was 0.047 < 0.5, hence this subject was correctly classified by the model in the low-score group; and the score obtained by the second was 0.906 > 0.5, and therefore this subject was also correctly classified by the model in the risk group. The classification of different subjects according to their probability of belonging to one or another group is represented in Figure 1. Most of the subjects of the risk group have been located in the area of scores between 0.5 and 1, while most of the subjects with low scores have been located in the area of scores between 0 and 0.5.

Furthermore, of particular note was the strong joint influence of the variables “mother’s limit setting” and “father’s support” on the possibility of belonging to the risk or low-score group, since when logistic regression analysis was applied, only inserting these factors as independent variables, 82.1% of subjects were correctly classified.

## 4. Discussion

The purpose of this study was to analyze which factors in the parenting practices and attitudes of fathers and mothers were linked with a higher or lower probability of obtaining a score in the risk area of the BASC in relation to depression.

If we focus on the previous comparison that has been carried out using ANOVA, all the variables considered could act as a good predictor of depression, because in all cases significant differences are obtained. We therefore agree with Roa and Del Barrio [33], and Richard de Minzi [24], in which high scores on the PCRI-M scales relate to a more appropriate parenting style that promotes a better adaptation in children.

Based on the model of parenting practices suggested by Darling and Steinberg [14], it is possible to identify family models or general patterns of parental behavior that would be directly related with depression in their children. Even accepting some methodological limitations like small group size, a wide age range, cross-sectional, using questionnaires and behavior reports taken from a single source of information, or the lack of information about some aspects of parents, like marital status or education level, the results obtained reflect major differences between the two groups. For nine out of every ten subjects included in the sample, a combination of low scores in support, satisfaction, communication, and autonomy granted by the father, and involvement and limit setting on the part of the mother, has a significant influence on the increased probability of scoring in the high-risk area of the BASC for depression. Due to its relevance, it is considered important to highlight the significance of limit setting on the part of the mother. This could be due to the fact that mothers spends more time with the children, so this practice affects the development of depression in the children more than in the case of fathers.

The model created is made up of a number of significant variables, but also included some variables that, although not significant, make an important contribution to the model’s predictive ability, increasing the number of correctly classified subjects. In this respect, limit setting—understood as the establishment of clear limits—acts as an excellent predictor of lower levels of depression in children, corroborating the affirmations of Yap and Jorm [20]. Another of the most relevant variables of this study is the support perceived by the parents. In this regard, as indicated Wilkinson et al. [26], a stressful context and the lack of support for parenting are very important risk factors for the development of disorders such as depression in the children.

Finally, we cannot ignore that these kinds of problems must be analyzed in terms of interaction, since although the family situation described might be a precursor to depression, these problems in children can also destabilize the family dynamic, generating inappropriate patterns of interaction with the parents.

## 5. Conclusions

Although the method used does not allow us to establish causal relationships, the information obtained has broad applications for interventions with families with depressed children, since the parents’ responses to the instruments utilized reveal patterns of behavior that can be modified in both parents and children. For this purpose, clear rules could be established and enforced through daily monitoring strategies in different tasks, being communicative with their children and involving all members of the family in the different parenting tasks to support a healthy work/life balance for both parents. In this sense, new treatments for intervening in children with behavioral problems and their families, like the Parent–Child Interaction Therapy (PCIT) can be applied. PCIT is based on the relationship between parents and children through play, creating new parental styles [36]. In addition, this therapy has been applied to childhood depression, finding several studies effective results before an early intervention [37,38].

Further research could be conducted to provide information about certain aspects yet to be clarified, such as the possible differences between the most important variables in the prediction of depression in young children and adolescents. Moreover, this study opens up a broad new avenue of research, through which other problems could be tackled, like behavioral problems or separation anxiety in childhood. Furthermore, other possible future studies could evaluate the effect of other influential factors such as those related to school environment and peers. This is interesting because, for children and adolescents, the influence of these relationships becomes increasingly important during the life cycle [39,40].

## Figures and Tables

**Table 1:** ANOVA between the two groups for parenting scales.

VARIABLES		High Risk	SD	Low Risk	SD	F
**Fathers**	Support	23.86	3.355	28.27	3.212	63.233 **
	Satisfaction	33.61	4.572	38.04	2.306	52.351 **
	Involvement	43.06	3.698	47.14	3.372	46.650 **
	Communication	28.03	2.944	30.86	3.245	29.176 **
	Limit setting	30.57	4.179	36.07	4.642	54.279 **
	Autonomy	24.40	3.038	28.10	2.783	56.462 **
	Role orientation	27.99	3.241	30.46	3.877	16.740 **
**Mothers**	Support	21.97	3.353	27.36	3.799	79.062 **
	Satisfaction	33.34	5.021	38.11	2.171	53.254 **
	Involvement	43.30	4.077	48.06	2.853	63.978 **
	Communication	28.77	3.576	31.39	2.901	22.565 **
	Limit setting	28.83	4.310	35.94	4.671	87.692 **
	Autonomy	24.30	3.364	27.96	3.127	44.381 **
	Role orientation	27.53	3.087	30.81	3.883	30.709 **

** *p* < 0.05.

**Table 2:** Variables included in the model.

VARIABLES	B	Standard Error	Wald	Degree of Freedom	*p*	Exp (B)	95% Confidence Interval Exp (B)	
							Lower	Higher
Father’s support	−0.286	0.103	7.804	1	0.005	0.751	0.614	0.918
Father’s satisfaction	−0.171	0.119	2.060	1	0.151	0.843	0.667	1.064
Father’s communication	−0.182	0.110	2.749	1	0.097	0.834	0.672	1.034
Father’s autonomy	−0.223	0.125	3.165	1	0.075	0.800	0.626	1.023
Mother’s involvement	−0.278	0.107	6.791	1	0.009	0.758	0.615	0.934
Mother’s limit setting	−0.251	0.077	10.691	1	0.001	0.778	0.670	0.904
Constant	45.780	8.400	29.704	1	0.000	7.620E19		
